# Supplementary material for: Divergent behavior amid convergent evolution: A case of four desert rodents learning to respond to known and novel vipers
Source: PLoS One. 2018 Aug 20;13(8):e0200672. doi: 10.1371/journal.pone.0200672 (PMC6101362; doi:10.1371/journal.pone.0200672)

S1. Fig. Interview chambers set-up. Images of the interview chamber setup: (A) As used in the experiment – Color coded (Blue- Horned Viper, Red- Sidewinder, and Green- Control. (B) Allenby’s gerbil out skirting a Saharan Horned Viper within a room of a chamber with the GUD patch filled with sand.

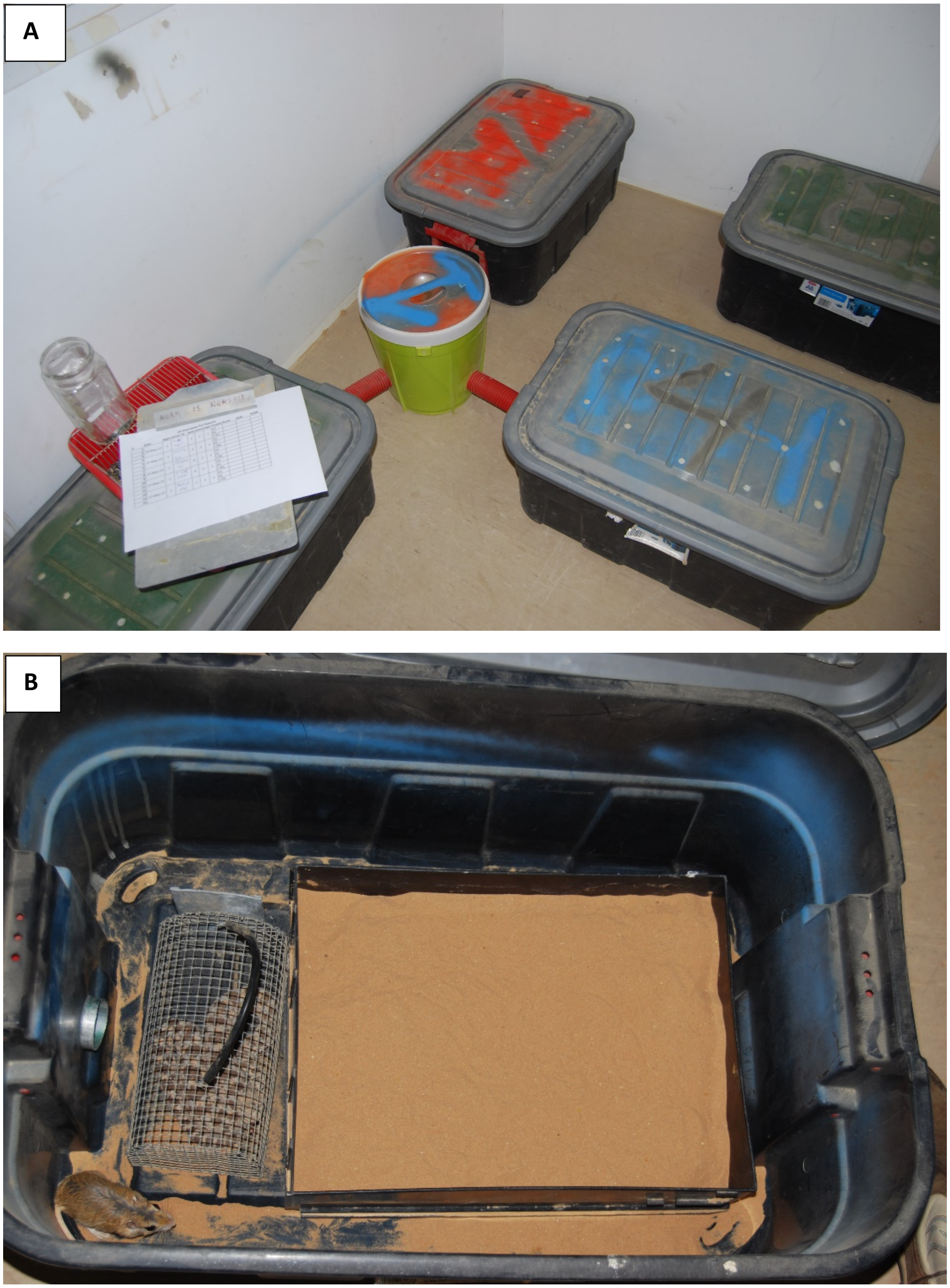

Supplement: S1 Fig — Images of the interview chamber setup: (A) As used in the experiment–Color coded (Blue- Horned Viper, Red- Sidewinder, and Green- Control. (B) Allenby’s gerbil out skirting a Saharan Horned Viper within a room of a chamber with the GUD patch filled with sand. (PDF) [file pone.0200672.s001.pdf]
