# Supplementary material for: Divergent behavior amid convergent evolution: A case of four desert rodents learning to respond to known and novel vipers
Source: PLoS One. 2018 Aug 20;13(8):e0200672. doi: 10.1371/journal.pone.0200672 (PMC6101362; doi:10.1371/journal.pone.0200672)

S2. Fig. Variations in foraging tenacity per species. Foraging tenacity (giving-up density ± SE) as a factor of the status of the predator (know or novel) to each of the four rodent species where the * stands for the snake with infra-red sensing pit organs. Abbreviations: GUD – giving-up density, CP -desert pocket mice, DM – Marriam's kangaroo rat, GA – Allenby's gerbil, GP – Egyptian gerbil.

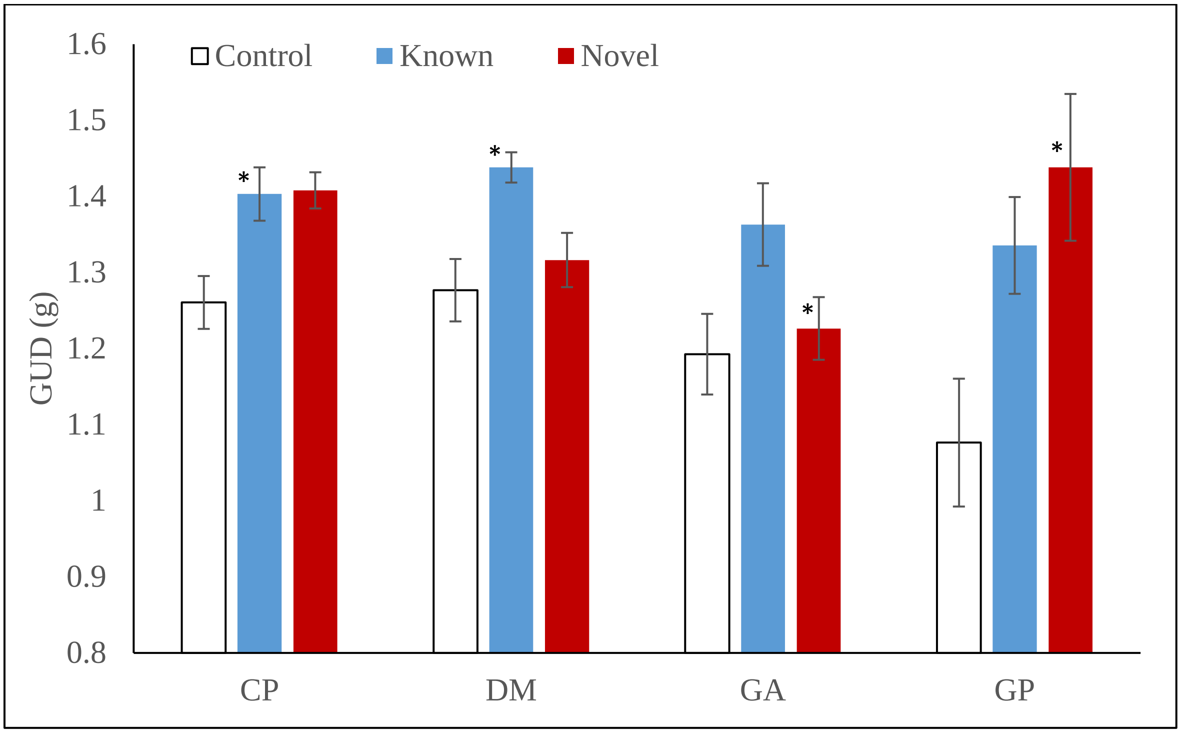

Supplement: S2 Fig — Foraging tenacity (giving-up density ± SE) as a factor of the status of the predator (know or novel) to each of the four rodent species where the * stands for the snake with infra-red sensing pit organs. Abbreviations: GUD–giving-up density, CP -desert pocket mice, DM–Marriam’s kangaroo rat, GA–Allenby’s gerbil, GP–Egyptian gerbil. (PDF) [file pone.0200672.s002.pdf]
