# Supplementary material for: Divergent behavior amid convergent evolution: A case of four desert rodents learning to respond to known and novel vipers
Source: PLoS One. 2018 Aug 20;13(8):e0200672. doi: 10.1371/journal.pone.0200672 (PMC6101362; doi:10.1371/journal.pone.0200672)
Supplement: S1 Table — Dates on which experiments took place: the variations in time are a result of the differences in sample sizes (post exposure only interviewing individuals who survived exposure to the vipers and owls and pre-exposure providing extra numbers to replace individuals that would be depredated during the exposures. (PDF) [file pone.0200672.s003.pdf]

S1. Table of Experimentation Dates. Dates on which experiments took place: the variations in time are a result of the differences in sample sizes (post exposure only interviewing individuals who survived exposure to the vipers and owls and pre-exposure providing extra numbers to replace individuals that would be depredated during the exposures.

Species	Pre-Exposure Interviews	Post-Exposure Interviews
GA	3-26/5/2011	3-9/8/2011
CP	2-5/7/2012	20-22/9/2012
DM	6-11/7/2012	8-10/11/2012
GP	27-29/5/2013	30/7/2013-2/8/2013
