# Supplementary material for: Divergent behavior amid convergent evolution: A case of four desert rodents learning to respond to known and novel vipers
Source: PLoS One. 2018 Aug 20;13(8):e0200672. doi: 10.1371/journal.pone.0200672 (PMC6101362; doi:10.1371/journal.pone.0200672)
Supplement: S2 Table — Random-forest output using as Giving-up densities as a dependent variable and testing for the effects of the rodent species, the snake treatments and the chorological sequence of interviews. Abbreviations: Node- connection point in the tree, Snake- snake treatment, GA- Allenby’s Gerbil, Sequence (pre-/ post-exposure), PV- Sidewinder Rattlesnake, GP- Greater Egyptian Gerbil, DM- Merriam’s Kangaroo Rat, HV- Saharan Horned Viper. (PDF) [file pone.0200672.s004.pdf]

S2 Table. Random-Forest Tree Structure Table. Random-forest output using as Giving-up densities as a dependent variable and testing for the effects of the rodent species, the snake treatments and the chorological sequence of interviews.

#	Child Node	Child Node	Node Size (N)	Node Mean	Node Variance	Split Variable	Split	Split
1	2	3	188	1.311193	0.084995	Species	GA	
2	4	5	70	1.260264	0.134589	Snake	CTRL	
4	6	7	25	1.107140	0.071674	Sequence	PRE	
6			15	1.083300	0.104403			
7			10	1.142900	0.020450			
5	8	9	45	1.345333	0.149280	Snake	PV	
8	10	11	21	1.270333	0.050358	Sequence	PRE	
10			15	1.267433	0.059383			
11			6	1.277583	0.027723			
9			24	1.410958	0.226607			
3	12	13	118	1.341404	0.053123	Snake	CTRL	
12			40	1.260180	0.051147			
13	14	15	78	1.383058	0.049018	Species	GP	DM
14	16	17	53	1.352292	0.062680	Snake	HV	
16			28	1.298500	0.084800			
17	18	19	25	1.412540	0.031035	Species	GP	
18			8	1.340938	0.055287			
19			17	1.446235	0.016074			
15	20	21	25	1.448280	0.013794	Sequence	PRE	
20	22	23	17	1.425118	0.018599	Snake	PV	
22			5	1.385300	0.049165			
23			12	1.441708	0.004928			
21			8	1.497500	0.000019			

Abbreviations: Node- connection point in the tree, Snake- snake treatment, GA- Allenby's Gerbil, Sequence (pre-/ post-exposure), PV- Sidewinder Rattlesnake, GP- Greater Egyptian Gerbil, DM-Merriam's Kangaroo Rat, HV- Saharan Horned Viper
